# MicroRNA childhood cancer catalog (M3Cs): a resource for translational bioinformatics toward health informatics in pediatric cancer

**DOI:** 10.1093/database/baac013

**Published:** 2022-03-18

**Authors:** Wafaa M Rashed, Fatima Adel, Mohamed A Rezk, Lina Basiouny, Ahmed A Rezk, Ahmed H Abdel-Razek

**Affiliations:** Research Department, Children’s Cancer Hospital Egypt, 1 Seket Al-Emam Street – El-Madbah El-Kadeem Yard – El-Saida Zenab, Cairo 11441, Egypt; Research Department, Children’s Cancer Hospital Egypt, 1 Seket Al-Emam Street – El-Madbah El-Kadeem Yard – El-Saida Zenab, Cairo 11441, Egypt; Armed Forces College of Medicine, Ehsan Abd El Kodous Street (From Al Khalifa Al Mamon Street), Cairo 55411, Egypt; Research Department, Children’s Cancer Hospital Egypt, 1 Seket Al-Emam Street – El-Madbah El-Kadeem Yard – El-Saida Zenab, Cairo 11441, Egypt; Armed Forces College of Medicine, Ehsan Abd El Kodous Street (From Al Khalifa Al Mamon Street), Cairo 55411, Egypt; Armed Forces College of Medicine, Ehsan Abd El Kodous Street (From Al Khalifa Al Mamon Street), Cairo 55411, Egypt

## Abstract

MicroRNA childhood Cancer Catalog (M3Cs) is a high-quality curated collection of published miRNA research studies on 16 pediatric cancer diseases. M3Cs scope was based on two approaches: data-driven clinical significance and data-driven human pediatric cell line models. Based on the translational bioinformatics spectrum, the main objective of this study is to bring miRNA research into clinical significance in both pediatric cancer patient care and drug discovery toward health informatics in childhood cancer. M3Cs development passed through three phases: 1. Literature Mining: It includes external database search and screening. 2. Data processing that includes three steps: (a) Data Extraction, (b) Data Curation and annotation, (c) Web Development. 3. Publishing: Shinyapps.io was used as a web interface for the deployment of M3Cs. M3Cs is now available online and can be accessed through https://m3cs.shinyapps.io/M3Cs/. For data-driven clinical significance approach, 538 miRNAs from 268 publications were reported in the clinical domain while 7 miRNAs from 5 publications were reported in the clinical & drug domain. For data-driven human pediatric cell line models approach, 538 miRNAs from 1268 publications were reported in the cell line domain while 211 miRNAs from 177 publications in the cell line & drug domain. M3Cs acted to fill the gap by applying translational bioinformatics general pathway to transfer data-driven research toward data-driven clinical care and/or hypothesis generation. Aggregated and well-curated data of M3Cs will enable stakeholders in health care to incorporate miRNA in the clinical policy.

Database URL:https://m3cs.shinyapps.io/M3Cs/

## Introduction

Although pediatric cancer diseases are rare, they represent a significant cause of both mortality and morbidity (1). Favorable outcomes of pediatric cancer diseases require effective risk assessment and early diagnosis followed by personalized treatment. By harnessing the power of early diagnosis and personalized treatments in pediatric cancer diseases, the overall healthcare system outcome will be improved, and the cost of therapy will be lowered.

MicroRNAs (miRNAs) are small RNA molecules (≈ 22 nucleotides) that belong to the class of noncoding RNA (2). They play an important role in posttranscriptional regulation of gene expression via mRNA degradation and/or translational repression (3). Research has accumulated a large body of evidence implicating many miRNAs in the diagnosis, prognosis and risk of hematological and solid tumors of childhood cancer (4–8). Using bioinformatics, there are about seven categories of online miRNA databases, however, none of which includes specialized miRNA databases for pediatric cancers (9).

Great advances in biomedical research (e.g. high-throughput sequencing and functional analysis) have led to voluminous data that needs proper processing into information then transformation into knowledge applied in the clinical setting. This will fill the gap between bench and bedside and enable clinicians to achieve favorable outcomes of pediatric cancer diseases. Translational Bioinformatics (TBI) is one of the biomedical research channels that can play an inevitable role in this aspect. Through a convergence of molecular bioinformatics, clinical informatics, genetics, biostatics and health informatics, recently many TBI databases have been established (10). Not only did these databases create new knowledge but also they provided novel insights into the disease genetic mechanism. Database for miRNA of pediatric cancer will structure the microRNA clinical utility knowledge in pediatric cancer and consequently, it may help in utilizing miRNA research findings into the clinical side.

**Figure 1.**
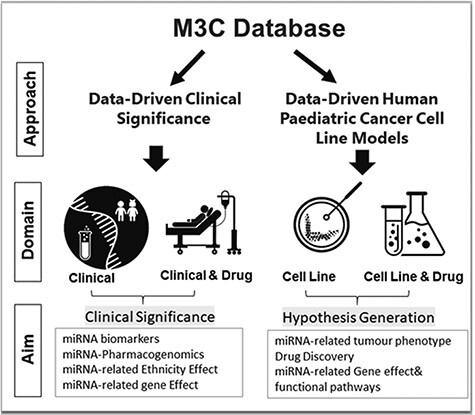
M3Cs approaches, domains and aims.

That is why we have launched MicroRNA Childhood Cancer Catalog (M3Cs). Based on the TBI spectrum, the principle of this platform is to bring miRNA research into clinical significance in both pediatric cancer patient care and drug discovery and toward health informatics in childhood cancer. This will ease the policymakers’ exploitation of pediatric cancer patients’ data to shift emphasis to prevention, allow the selection of optimal diagnosis and therapy and reduce trial and error prescribing as well as reviving drugs that failed early in clinical trials. Additionally, it will help scientists for more hypothesis generation. To achieve this goal, we have utilized two approaches: data-driven clinical significance and data-driven human pediatric cell line models (Figure 1).

## Method

**Figure 2.**
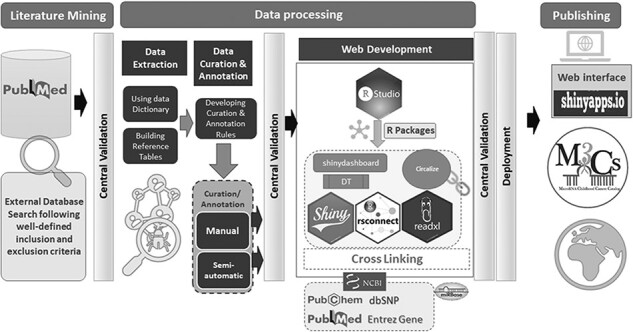
The overall workflow of M3Cs phases.

M3Cs development passed with three phases with central validation at the end of each phase (Figure 2).

### Phase 1: Literature mining: it included external database search and screening

M3Cs team was responsible for searching PubMed using the following search terms: {[(microRNA) OR miRNA] OR miR} AND ‘Disease name’. We have included the most common pediatric cancer diseases: Acute Lymphoblastic Leukemia (ALL), Acute Myeloid Leukemia (AML), Adrenocortical Tumor (ACT), Brain Tumor, Chronic Myelogenous leukemia (CML), Ewing Sarcoma (ES), Hepatoblastoma, Hodgkin disease, Juvenile myelomonocytic leukemia (JMML), Langerhans cell histiocytosis (LCH), Neuroblastoma (NB), Non-Hodgkin lymphoma (NHL), Osteosarcoma (OS), Renal tumor, Retinoblastoma (RB) and Rhabdomyosarcoma (RMS). Screening of search results was based on the inclusion and exclusion criteria.

**For data-driven clinical significance approach**, the inclusion criteria for studies are: (i) Patients with age less than 18 years; (ii) Publication with data about miRNA-related-disease diagnosis, -disease prognosis or -disease risk. For publications of prognostic miRNAs, survival results were measured, and it should include at least one survival curve [overall survival (OS), disease-free survival (DFS), recurrence/relapse-free survival (RFS), progression-free survival (PFS) and metastasis-free survival (MFS)] with or without HRs/95% CIs. MiRNA studies were excluded if: (i). Age of patients was not identified or above 18 years old; (ii). Reports or reviews or letters without primary data; (iii). Non-English publication; (iv). Retracted papers.

**For data-driven human pediatric cell line models approach,** the inclusion criteria of miRNA studies are (i) Human cell line of pediatric origin (<18 years) with verification from Cellosaurus (11) (species origin and age at sampling); (ii) Publication investigated the miRNA effect on the tumor phenotype. MiRNA studies were excluded if: (i) Data from animal models; (ii) Reports, reviews or letters without primary data; (iii) Non-English publications; (iv) Retracted papers. Central validation is the safeguard for M3Cs. Centralization of data validation decreased variability and ensured a database of high quality. The primary role of central validation at the end of phase 1 was to revise all included and excluded studies using the predefined criteria. Disputes regarding including or excluding studies have been resolved by discussion with the whole team.

### Phase 2: Data processing that included three steps: (i) Data extraction, (ii) Data curation & annotation, (iii) Web development

#### Step 1- Data extraction

**Figure 3.**
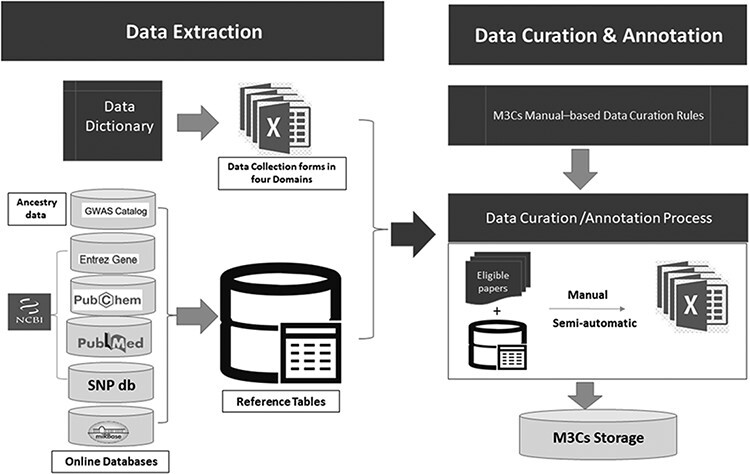
Detailed description of both data extraction and data curation/annotation as a part of data processing.

M3Cs is based on two main approaches: data-driven clinical significance approach and data-driven human pediatric cell line model approach. Each approach has two specific domains. For data-driven clinical significance approach, there is ‘clinical domain’ and ‘clinical & drug domain’. For data-driven human pediatric cell line model approach, there is ‘cell line domain’ and ‘cell line & drug domain’. Data dictionary for each domain was developed with the standardized data definitions and formats for the collection (Supplementary Tables S1–S4). Data collection forms were developed for each domain containing a recommended set of variables of data elements. These data collection forms were used to collect research data from each eligible study for the M3Cs database (Figure 3). Many online databases were used for reporting of some variables [e.g. miRBASE (12), Entrez gene (13), PubChem (14), dbSNP (15) and PubMed (16)]. Also, in the data-driven clinical significance approach, we followed GWAS catalog standard method of reporting ancestry data (17). To reduce time-consuming of data curation and annotation step, reference tables were built in the data extraction step.

#### Step 2- Data curation & annotation

Data curation & annotation were done manually and semiautomatic by well-trained scientists. They followed specific curation rules based on the M3Cs manual. Using reference tables, all eligible studies were curated in the domain-specific collection form. The data derived from eligible papers were linked to microRNA entry. All collection forms were stored on M3Cs storage database on google drive which is very secure and easily accessible by the M3Cs team. At the end of this phase, **central validation** was used to check both the quality and accuracy of manually curated and annotated data.

#### Step 3- Web development

M3Cs is a web application that has been developed by R programming language (version 4.0.2) and RStudio was used as an interface (https://www.r-project.org) and Shiny framework was used for deployment of the application (https://CRAN.R-project.org/package=shiny). The data of each domain were presented using the DT package, while the miRNA plots were depicted with the circlize package. We have used crosslink to ease access to other websites. The rsconnect package was used for deployment of the shiny application. At the end of this phase, **central validation** was responsible for checking high-quality cross-links.

#### Phase 3: Publishing

Shinyapps.io platform was used as a web interface for deployment of M3Cs application. Shinyapps.io platform is secure by design and allows M3Cs shiny application to run in its own protected environment.

## Result

M3Cs can be searched using the web interface https://m3cs.shinyapps.io/M3Cs/. The homepage of M3Cs includes a disclaimer that gives important notices for users related to many issues: the content, the medical information and advice, the endorsement, the intellectual property, as well as the external links. Out of 6542 studies screened from PubMed between 2002 and the end of 2020, 1872 studies were included in the M3Cs. The total number of included and excluded studies in each disease in M3Cs were summarized in Table 1. In phase 1 (literature mining), we did not find any miRNA-related LCH study in pediatric cancer patients.

**Table 1.** Summary of the total number of included and excluded studies for each disease in the overall phases of M3Cs

Serial	Disease	Included	Excluded	Total Screened
1	Acute Lymphoblastic Leukemia	108	227	**335**
2	Acute Myeloid Leukemia	115	911	1026
3	Adrenocortical Tumors	1	73	74
4	Brain Tumors	78	233	**311**
5	Chronic Myelogenous Leukemia	4	314	**318**
6	Ewing Sarcoma	22	50	**72**
7	Hepatoblastoma	149	51	**200**
8	Hodgkin Disease	1	226	**227**
9	Juvenile Myelomonocytic leukemia (JMML)	3	5	**8**
10	Langerhans cell Histiocytosis (LCH)	0	0	**0**
11	Neuroblastoma	160	416	**576**
12	Non-Hodgkin Lymphoma (NHL)	38	622	**660**
13	Osteosarcoma	1084	270	1354
14	Rhabdomyosarcoma (RMS)	31	102	**133**
15	Renal Tumor	14	1131	**1145**
16	Retinoblastoma	64	39	**103**
Total		1872	4670	**6542**

**Figure 4.**
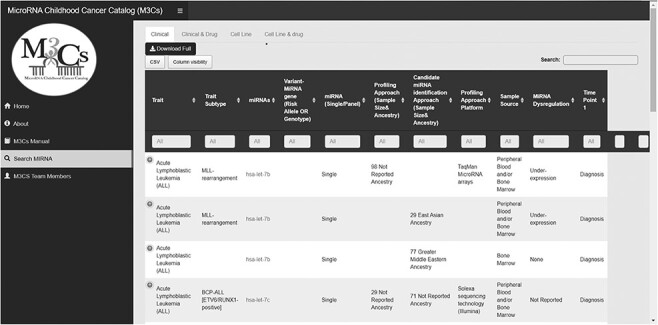
Screenshot of search miRNA page in M3Cs.

The M3Cs manual page includes a data dictionary for each M3Cs domain (Clinical, Clinical & Drug, Cell line and Cell line & Drug) to give the definitions of different variables. The main M3Cs search page includes the four domains. M3Cs allows retrieving information of candidate miRNA in each domain. Also, it can be searched with different possible searches due to using filter tables in each domain (Figure 4).

### I—Data-driven clinical significance approach

In the clinical domain, miRNA role as a probable diagnostic, prognostic, predictive biomarker along with miRNA-Polymorphism value as a probable risk factor for pediatric cancer trait and trait subtype has been reported.

M3Cs helps to identify reported miRNAs that can be used as a biomarker panel (a group of miRNAs or miRNAs and genes). M3Cs determines the study design approach that includes a profiling approach and then a candidate approach as a validation or profiling approach only or a candidate approach only. This was achieved via identification of the platform used (e.g. miRNA arrays only or qRT-PCR only or both). In the clinical & drug domain, M3Cs reports clinical studies related to miRNAs-associated drug toxicity in pediatric cancer traits and trait subtypes. Variants in miRNA gene and drug toxicity associated with this have been reported. In both domains, we provided the sample size for each distinct group of the sample included in the analysis and assigned ancestry category for each group. Pediatric patients’ ancestry was reported following the GWAS catalog framework (17). In addition, miRNA dysregulation and their tested direct downstream target gene(s) have been reported. All statistical significant parameters described by the original study have been included by M3Cs e.g. hazard ratio, covariates, odds ratio, relative risk, sensitivity and specificity.

### II—Data-driven human pediatric cancer cell line model approach

In the Cell Line domain, M3Cs included most human pediatric cell line studies that identify the effect of dysregulated miRNA on the tumor phenotype in each pediatric cancer trait and trait subtype to differentiate between miRNAs that act as a tumor suppressor or an oncogene. In the Cell Line & Drug domain, studies examining the drug effect on miRNA expression in each pediatric cancer trait and trait subtype have been included. In both domains, direct downstream target genes associated with this miRNA along with the underlying affected downstream pathways have been reported. Publication title, PubMed and Notes were the three fixed variables included in all domains.

If M3Cs team missed specific publications about microRNA in pediatric cancer, users are welcome to send us using M3Cs contact email (mirna.childhood.cancer.catalog@gmail.com) and the M3Cs team is pleased to add them to the M3Cs. Using the same email, M3Cs users can contact M3Cs to draw circos plots to visualize miRNA.

## Discussion

In the new era of innovative personalized medicine, novel approaches are rapidly flourishing toward treatment specifications and the use of targeted precision therapy. Precision oncology and the identification of targetable signatures of tumors through molecular profiling ensures that cancer treatment is specifically targeted and tailored to each distinctive tumor. MicroRNAs can act as remarkable signatures for tumor cells helping to promptly diagnose, predict outcomes and potentially act as a therapeutic target for different tumors. Also, genetic profiling of tumors to detect genomic, epigenomic and transcriptomic alterations ensures a clear understanding of tumor pathogenesis and identification of target binding sites. MicroRNAs have a great potential to be exploited in the field of precision oncology due to their high specificity, sensitivity and stability in patients’ body fluids as well as their wide availability and simple quantification.

M3Cs is the first specialized miRNA pediatric cancer database. M3Cs utilized two approaches: data-driven clinical significance and data-driven human pediatric cell line model approaches. In data-driven clinical significance, M3Cs assembled studies that investigate the potential role of miRNA as a diagnostic, prognostic, and predictive biomarker in each pediatric cancer disease. In addition, it promotes researchers to use data to investigate a new biomarker panel and to pursue more meta-analysis studies. Furthermore, it can be used as a springboard for drug discovery and gene therapy. Moreover, it reports miRNA-Polymorphism that affects drug response (miRNA Pharmacogenomics). This will pay attention to miRNA pharmacogenomics to be used in contemporary treatment protocols and consequently more research in this promising field.

Furthermore, M3Cs reports clinical studies with dysregulated miRNAs and their tested direct target gene(s). It enables researchers to fuse diagnostics with targeted therapy as personalized medicines to improve both the management and outcome of many pediatric cancer diseases.

In data-driven human pediatric cell line model approach, M3Cs sheds light on miRNA value as a targeted therapy. Developing strategies to replenish tumor-suppressive miRNAs (miRNA replacement) or supprese oncomiRs (anti-miRNAs) can be utilized as therapeutic modulation of miRNAs. Besides, developing chemical modification and delivery systems for these miRNA-based therapeutics have been introduced *in vivo* cancer models (18). All these factors will encourage researchers to develop *in vivo* models to treat different types of pediatric cancers.

Also, M3Cs will help computer scientists to use the curated data in creating simulation for easy biomarker discovery and modeling of any biological system using big data.

M3Cs have developed variables of datasets that can be used by the journal editors and reviewers to evaluate submitted miRNA articles. We have excluded many publications that include missing parts in analysis that can be included upon reviewers’ request. Standardization of reporting in miRNA publications will help researchers to advance miRNA research and applications in terms of increasing the number of evidence.

There will be a half-annual update of M3Cs that will include adding new publications and removing any retracted paper from M3Cs records.

### Future plan

M3Cs survey has been developed to help users to send their feedback about M3Cs design, content and ease of search. M3Cs version 2 (M3Cs v.2) will take in consideration M3Cs users’ feedback as well as the inclusion of more pediatric cancer types and other features.

M3Cs has certain advantages over other available miRNA databases (9, 19). The M3Cs construction and the involved variables are unique to M3Cs. M3Cs focuses only on miRNA studies in pediatric cancer. This enabled M3Cs team to organize data into specific domains under clinical and human pediatric cell line approaches in one catalog. In all domains, M3Cs reported the potential role of single or panel miRNAs with specification of the pediatric cancer subtype. Also, in the clinical domain, M3Cs included variables about the ethnicity of study subjects, the potential role of miRNAs as a diagnostic, prognostic and/or predictive biomarker in each pediatric cancer subtype. This is in addition to other variables that was reported to support this potential role. In addition, M3Cs was constructed using shinyapps.io platform and allow users to use it without prior database knowledge. Also, it allows exporting of data and custom queries and it includes database links for more information about specific M3Cs data. Moreover, it offers visualization of miRNAs as Circos plot as a free service to all users. Due to lack of fund, M3Cs has specific limitation. It did not include data of animal model in this M3Cs version but M3Cs team will consider adding animal model approach as a third approach in M3Cs version 2. This is after a good construction of the underlying domains and their variables. In M3Cs, we followed the general pathway of TBI of data collection, storage, analysis, retrieval and interpretation. We acted to fill the gap by applying TBI which simply transfers data-driven research toward data-driven clinical care and/or hypothesis generation. Information that is aggregated and well-curated will enable stakeholders in health care to incorporate miRNA in the clinical policy to be used by clinicians in their clinics.

## Supplementary Material

baac013_Supp

## References

[1] Johnston W.T. , Erdmann F., Newton R. et al. (2021) Childhood cancer: estimating regional and global incidence. *Cancer Epidemiol.*, 71, 101662. 10.1016/j.canep.2019.101662 31924557

[2] Rashed W.M. , Hamza M.M., Matboli M. et al. (2019) MicroRNA as a prognostic biomarker for survival in childhood acute lymphoblastic leukemia: a systematic review. *Cancer Metastasis Rev.*, 38, 771–782. 31807971 10.1007/s10555-019-09826-0

[3] Catalanotto C. , Cogoni C. and Zardo G. (2016) MicroRNA in control of gene expression: an overview of nuclear functions. *Int. J. Mol. Sci.*, 17, 1712. 10.3390/ijms17101712 PMC5085744 27754357

[4] Leichter A.L. , Sullivan M.J., Eccles M.R. et al. (2017) MicroRNA expression patterns and signalling pathways in the development and progression of childhood solid tumours. *Mol. Cancer*, 16, 15. 10.1186/s12943-017-0584-0 PMC5248531 28103887

[5] Gulino R. , Forte S., Parenti R. et al. (2015) MicroRNA and pediatric tumors: future perspectives. *Acta Histochem.*, 117, 339–354. 25765112 10.1016/j.acthis.2015.02.007

[6] Carvalho de Oliveira J. , Molinari Roberto G., Baroni M. et al. (2018) MiRNA dysregulation in childhood hematological cancer. *Int. J. Mol. Sci.*, 19.doi: 10.3390/ijms19092688. PMC6165337 30201877

[7] Smith C.M. , Catchpoole D. and Hutvagner G. (2019) Non-coding RNAs in pediatric solid tumors. *Front. Genet.*, 10, 798. 10.3389/fgene.2019.00798 PMC6764412 31616462

[8] Murray M.J. , Raby K.L., Saini H.K. et al. (2015) Solid tumors of childhood display specific serum microRNA profiles. *Cancer Epidemiol. Biomarkers Prev.*, 24, 350–360. 25416717 10.1158/1055-9965.EPI-14-0669 PMC4340540

[9] Shaker F. , Nikravesh A., Arezumand R. et al. (2020) Web-based tools for miRNA studies analysis. *Comput. Biol. Med.*, 127, 104060. 10.1016/j.compbiomed.2020.104060 33096299

[10] Singh O. , Chang N.-W., Dai H.-J. et al. (2019) Translational bioinformatics databases. In: *Encyclopedia of Bioinformatics and Computational Biology*, Vol. 1058–1062. Elsevier.doi: 10.1016/B978-0-12-809633-8.20303-8.

[11] Bairoch A. (2018) The cellosaurus, a cell-line knowledge resource. *J. Biomol. Tech.*, 29, 25–38. 29805321 10.7171/jbt.18-2902-002 PMC5945021

[12] Kozomara A. , Birgaoanu M. and Griffiths-Jones S. (2019) miRBase: from microRNA sequences to function. *Nucleic Acids Res.*, 47, D155. 10.1093/nar/gky1141 PMC6323917 30423142

[13] Brown G.R. , Hem V., Katz K.S. et al. (2015) Gene: a gene-centered information resource at NCBI. *Nucleic Acids Res.*, 43, D36–D42. 25355515 10.1093/nar/gku1055 PMC4383897

[14] Kim S. , Chen J., Cheng T. et al. (2020) PubChem in 2021: new data content and improved web interfaces. *Nucleic Acids Res.*, 49, D1388–D1395. 10.1093/nar/gkaa971 PMC7778930 33151290

[15] Sherry S.T. , Ward M.-H., Kholodov M. et al. (2001) dbSNP: the NCBI database of genetic variation. *Nucleic Acids Res.*, 29, 308–311. 11125122 10.1093/nar/29.1.308 PMC29783

[16] Fiorini N. , Canese K., Starchenko G. et al. (2018) Best match: new relevance search for PubMed. *PLoS Biol.*, 16, e2005343. 10.1371/journal.pbio.2005343 PMC6112631 30153250

[17] Morales J. , Welter D., Bowler E.H. et al. (2018) A standardized framework for representation of ancestry data in genomics studies, with application to the NHGRI-EBI GWAS Catalog. *Genome Biol.*, 19, 21. 10.1186/s13059-018-1396-2 PMC5815218 29448949

[18] Rupaimoole R. and Slack F.J. (2017) MicroRNA therapeutics: towards a new era for the management of cancer and other diseases. *Nat. Rev. Drug Discov.*, 16, 203–222. 28209991 10.1038/nrd.2016.246

[19] Mar-Aguilar F. , Rodríguez-Padilla C. and Reséndez-Pérez D. (2016) Web-based tools for microRNAs involved in human cancer. *Oncol. Lett.*, 11, 3563–3570. 27284356 10.3892/ol.2016.4446 PMC4887920

